# Emerging Role of Schwann Cells in Neuropathic Pain: Receptors, Glial Mediators and Myelination

**DOI:** 10.3389/fncel.2019.00116

**Published:** 2019-03-27

**Authors:** Zhongya Wei, Ying Fei, Wenfeng Su, Gang Chen

**Affiliations:** ^1^Key Laboratory of Neuroregeneration of Jiangsu and Ministry of Education, Co-innovation Center of Neuroregeneration, Nantong University, Nantong, China; ^2^Department of Anesthesiology, Affiliated Hospital of Nantong University, Nantong, China

**Keywords:** Schwann cells, neuropathic pain, receptors, glial mediators, myelination

## Abstract

Neuropathic pain caused by nerve injury or disease remains a major challenge for modern medicine worldwide. Most of the pathogenic mechanisms underlying neuropathic pain are centered on neuronal mechanisms. Accumulating evidence suggests that non-neuronal cells, especially glial cells, also play active roles in the initiation and resolution of pain. The preponderance of evidence has implicated central nervous system (CNS) glial cells, i.e., microglia and astrocytes, in the control of pain. The role of Schwann cells in neuropathic pain remains poorly understood. Schwann cells, which detect nerve injury and provide the first response, play a critical role in the development and maintenance of neuropathic pain. The cells respond to nerve injury by changing their phenotype, proliferating and interacting with nociceptive neurons by releasing glial mediators (growth factors, cytokines, chemokines, and biologically active small molecules). In addition, receptors expressed in active Schwann cells have the potential to regulate different pain conditions. In this review article, we will provide and discuss emerging evidence by integrating recent advances related to Schwann cells and neuropathic pain.

## Introduction

Neuropathic pain is a typically persistent and intractable type of chronic pain. This condition is not a symptom of a disorder but a pathological state caused by a primary lesion or dysfunction in the nervous system (Backonja, 2003). It is well known that neuropathic pain is an expression of neuroplasticity and arises from both the peripheral nervous system (PNS) and the central nervous system (CNS).

Past research on neuropathic pain has focused mostly on the role of neurons (Ji et al., 2003; Chen et al., 2016, 2017). Following a nerve injury, both peripheral and central sensitization act as important disease mechanisms, including sensitization and hyperexcitability of primary sensory neurons as well as enhanced excitatory synaptic transmission or reduced inhibitory synaptic transmission in the neurons of the CNS (Gold and Gebhart, 2010; Kuner, 2010). In parallel to the changes in the activity of neuronal systems, non-neuronal cells, especially glial cells, are increasingly recognized as important in the development and maintenance of neuropathic pain (Ji et al., 2013, 2016). Regarding the glial cells in the CNS, both astrocytes and microglia have well-documented roles in the regulation of neuropathic pain, primarily in the spinal cord and brain (Ji et al., 2013, 2016; Chen et al., 2014, 2018).

**Figure 1:**
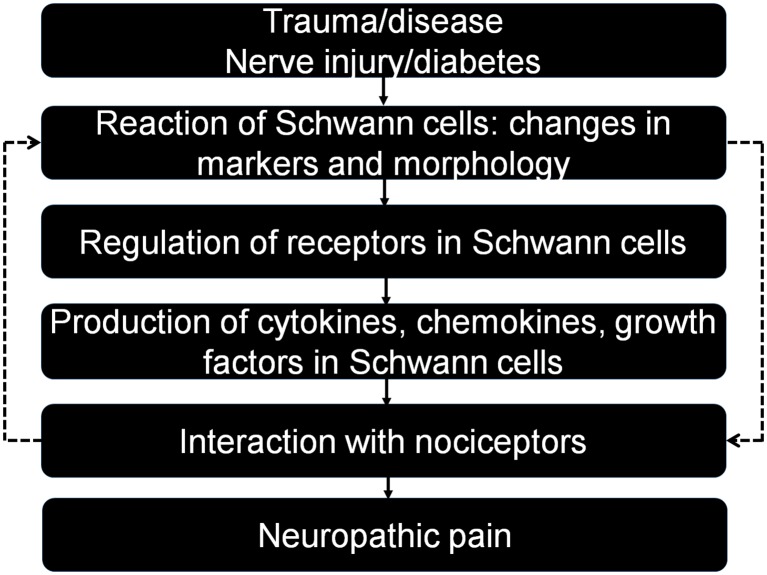
Interactions between Schwann cells and neurons in different pain conditions after trauma and disease. Note that Schwann cells can modulate neuropathic pain in different directions by producing relevant mediators that act on specific receptors. Activated neurons also have a feedback effect on Schwann cells, completing an interaction loop.

The glial cells of the PNS primarily include Schwann cells and satellite glial cells. The satellite glial cells surrounding the somata of dorsal root ganglion (DRG) neurons are activated prior to central glial cells after nerve injury and play a critical role in the development of neuropathic pain (Jasmin et al., 2010; Ji et al., 2013). Schwann cells not only physically support the long axons but also release a variety of growth factors to nourish and myelinate the large associated axons (Chen et al., 2012; Kidd et al., 2013; Su et al., 2016). After sciatic nerve injury, activated Schwann cells undergo dramatic changes in response, including phenotype modulation, proliferation, migration and release of numerous factors, which eventually promote nerve regeneration (Scheib and Höke, 2013). It is well known that nerve injury is of utmost importance in the generation of neuropathic pain. Thus, Schwann cells play a key role in the study of neuropathic pain, but little is known about how these cells regulate this condition. In this review article, we will discuss and update the current knowledge of how Schwann cells modulate neuropathic pain, and we will provide an improved understanding of the underlying mechanisms (Figure 1).

## Schwann Cells and Their Multipotency

Schwann cells, the most abundant glial cells in the PNS, include two major phenotypes: myelinating Schwann cells and non-myelinating Schwann cells. Both types originate from embryonic Schwann cell precursors derived from the neural crest. Initially, these cells surround the external margins of the axon bundles (Kidd et al., 2013). Schwann cells support axonal outgrowth during this stage by producing a variety of growth factors, such as nerve growth factor (NGF), glial cell line-derived neurotrophic factor (GDNF), and brain-derived neurotrophic factor (BDNF; Kidd et al., 2013). While maturing and interacting with axons, myelinating Schwann cells wrap larger axons at a one-to-one ratio to produce a myelin sheath, while nonmyelinating Schwann cells embed smaller axons, forming a Remak bundle (Kidd et al., 2013).

After nerve injury, the former myelinating cells degrade their myelin and become a class of nonmyelinating cells, repair Schwann cells, which regain their developmental potential, including the capacity for proliferation, growth factor production, sorting, or sprouting and myelination if they receive the suitable neuronal signals (Griffin and Thompson, 2008; Scheib and Höke, 2013). Importantly, signaling systems that are crucial for the development of the Schwann cell lineage, but they have no or little role in the generation of repair Schwann cells and nerve regeneration (Jessen and Arthur-Farraj, 2019). This encourages the view that the different phenotype of Schwann cells and their transitions are likely to play a different role in regulating neuropathic pain, but it still awaits the selective tools to control the particular cells and reveal their specific role in the process of neuropathic pain. Notably, multiple receptors, channels and active molecules are altered or activated in different neuropathic pain conditions. Glial mediators, including cytokines, chemokines and growth factors are produced and secreted from activated Schwann cells.

## Receptors, Channels and Active Molecules of Schwann Cells Mediate Neuropathic Pain

As shown in Table 1, multiple receptors and channels are expressed in Schwann cells and regulated in different pain conditions. Although these molecules are not released, they activate intracellular signaling to release growth factors, cytokines, and chemokines that regulate pain states (Table 2).

**Table 1:** Regulation of receptors and active molecules in Schwann cells in neuropathic pain.

Molecule	Classification	Change after nerve injury	References
P2X4R	Receptor	↑	Su et al. (2019)
P2X2/3R	Receptor	↑	Zhang et al. (2018)
P2X7R	Receptor	–	Faroni et al. (2014b)
TLR2	Receptor	↑	Boivin et al. (2007) and Lee et al. (2013)
LRP1	Receptor	↓	Campana et al. (2006a) and Orita et al. (2013)
TRPA1	Channel	↑	De Logu et al. (2017)
LPA1 R	Receptor	↑	Inoue et al. (2004)
HCAR2	Receptor	↑	Boccella et al. (2019)
GABA-B	Receptor	–	Faroni et al. (2014a) and Magnaghi et al. (2014)
MHC-II	Antigen	↑	Hartlehnert et al. (2017)
ErbB	Receptor	–	Chen et al. (2006)
gp120	Glycoprotein	–	Keswani et al. (2006)

*Purinergic receptors: P2X4R, P2X2/3R, P2X7R; TLR2, Toll-like receptor 2; LRP1, LDL receptor-related protein 1; TRPA1, Transient receptor potential Ankyrin 1; LPA1 R, Lysophosphatidic acid 1 receptor; HCAR2, Hydroxyl carboxylic acid receptor type 2; GABA-B, γ-aminobutyric acid type B receptor; MHC-II, Class II major histocompatibility complex; ErbB, Epidermal growth factor receptor; gp120, HIV-1 envelope glycoprotein. Symbols: ↑ denotes upregulation or activation; ↓ denotes downregulation or inactivation; – denotes data unavailable*.

**Table 2:** Regulation of cytokines, chemokines, and growth factors in Schwann cells in neuropathic pain.

Mediator	Classification	Change after nerve injury	References
TNF-α	Proinflammatory cytokine	↑	Scholz and Woolf (2007) and Sacerdote et al. (2008)
IL-1	Proinflammatory cytokine	↑	Martucci et al. (2008)
IL-6	Proinflammatory cytokine	↑	Martucci et al. (2008)
IL-10	Anti-inflammatory cytokine	↓	Franchi et al. (2012) and Wang et al. (2012)
Epo	Anti-inflammatory cytokine	↑	Keswani et al. (2004) and Campana et al. (2006b)
COX2	Chemokine	↑	Takahashi et al. (2004)
MCP-1	Chemokine	↑	Toews et al. (1998) and De Logu et al. (2017)
BDNF	Growth factor	↑	Yajima et al. (2005) and Su et al. (2019)
ATP	Messenger molecule	↑	Martucci et al. (2008)

*TNF-α, Tumor necrosis factor-α; IL-1, IL-6, IL-10, Interleukin related cytokine; Epo, Erythropoietin; COX-2, Cyclooxygenase-2; MCP-1, Monocyte chemoattractant protein-1; BDNF, Brain derived neurotrophic factor; ATP, Adenosine triphosphate. Symbols:↑denotes upregulation or activation; ↓denotes downregulation or inactivation*.

Adenosine triphosphate (ATP) is an important molecule in the process of pain information transmission (Kuan and Shyu, 2016). This substance modulates glial activation primarily *via* activating the P2X ion channel receptor and the G-protein-coupled receptor (GPCR)-coupled receptor P2Y (Lecca et al., 2012). Although both P2X and P2Y receptors are expressed in Schwann cells, accumulating evidence shows that P2X receptors play a critical role in the regulation of neuropathic pain (Mayer et al., 1998). mRNAs for all P2X receptor subtypes are detectable in Schwann cells, with P2X4–7 receptors being highly expressed (Su et al., 2019). *In vivo* and *in vitro* experiments have revealed that the expression of the P2X4 receptor is markedly upregulated in Schwann cells of injured nerves. Blocking the P2X4 receptor in microglia can reverse established pain hypersensitivity after nerve injury, and the development of pain hypersensitivity after nerve injury is prevented in P2X4 receptor knockout mice (Tsuda et al., 2003; Ulmann et al., 2008). However, we did not observe increased pain hypersensitivity in mice with specific overexpression of the P2X4 receptor in Schwann cells compared with the control group (Su et al., 2019). While the expression of P2X7 receptor in Schwann cells and Schwann cell-like adipose-derived stem cells has been found to contribute to ATP-induced cell death (Faroni et al., 2013). Notably, the study from P2X7 knockout mice has revealed that P2X7 knockout nerves possess more unmyelinated axons containing a higher number of Remak bundle, which increase nociception (Faroni et al., 2014b). Another report regarding Schwann cells involved in regulating neuropathic pain shows that the transplantation of microencapsulated Schwann cells can alleviate neuropathic pain by inhibiting P2X2/3 receptor overexpression in sciatic nerve injury (Zhang et al., 2018). The underlying mechanism of upregulation of P2X receptors in nerve injury is centered on the enhancement of BDNF release (Ulmann et al., 2008; Su et al., 2019), while the other mechanisms are still unclear.

Toll-like receptors (TLRs) are known to regulate innate immunity and have been strongly linked to the activation of glial cells (Nicotra et al., 2012). TLR2, 3 and 4 are highly expressed in Schwann cells at both the mRNA and protein levels (Lee et al., 2013). Lee et al. (2013) found that necrotic sensory neurons induced the release of proinflammatory mediators such as tumor necrosis factor-α (TNF-α) and iNOS by cultured rat Schwann cells from wild-type mice but not those from TLR2 knockout mice, suggesting that Schwann cells are activated through TLR2 recognition of damage-associated molecular patterns (DAMPs) during nerve injury. Notably, an *in vivo* study by Boivin et al. (2007) demonstrated that the Wallerian degeneration and expression of proinflammatory molecules induced by nerve injury were severely impaired in TLR2-knockout mice. Strikingly, the male specificity of the involvement of spinal TLR4 in neuropathic pain suggests a sex difference in TLR4 and microglial signaling (Sorge et al., 2011). Despite the lack of evidence that Schwann cells are involved in the sex difference in TLR signaling in neuropathic pain, any research to develop a TLR-antagonist analgesic or characterize a *Tlr* mutation must take the effect of sex differences into account if such information is available.

The endocytic transmembrane receptor known as LDL receptor-related protein 1 (LRP1) is a potent regulator of Schwann cells that orchestrates many of the physiological changes and the activation of Schwann cells after injury (Campana et al., 2006a; Mantuano et al., 2011). When Schwann cells lose LRP1 function through a cell-type-specific deletion in scLRP1^−/−^ mice or through an antagonist of the receptor-associated protein, both the survival and the function of those cells are compromised (Campana et al., 2006a; Orita et al., 2013). Prior to nerve injury, *LRP1* gene deletion in Schwann cells resulted in the activation of regeneration-associated genes in DRG neurons and the potential to cause chronic pain. Although the presence of abnormal Schwann cells in scLRP1^−/−^ mice primed injured DRG neurons to develop neuropathic pain, nerve repair in scLRP1^−/−^ mice was associated with abnormalities in ultrastructure and primarily in Remak bundles (Orita et al., 2013; Boivin et al., 2007).

It is well known that transient receptor potential ankyrin 1 (TRPA1) channels are highly expressed by a subpopulation of primary sensory neurons (Story et al., 2003). TRPA1 has been shown to mediate mechanical hypersensitivity in different types of neuropathic pain, including those induced by peripheral nerve injury (Eid et al., 2008). In 2017, TRPA1 was found to be expressed in Schwann cells both in cultures and in the sciatic nerve trunk (De Logu et al., 2017). The same study found that TRPA1 silencing in nociceptors attenuated mechanical allodynia without affecting macrophage infiltration, whereas TRPA1 silencing in Schwann cells reduced both mechanical allodynia and neuroinflammation. Activation of Schwann cell TRPA1 induces and maintains macrophage infiltration to the injured nerve and sends paracrine signals to activate TRPA1 in ensheathed nociceptors to sustain mechanical allodynia (De Logu et al., 2017).

Lysophosphatidic acid (LPA) is a bioactive lipid that interacts with specific GPCRs (Ishii et al., 2004). At least three specific LPA receptors are expressed in Schwann cells, and this cell type is known to primarily express the LPA1 receptor (Weiner et al., 2001). The LPA1 receptor is upregulated following sciatic nerve injury, and Schwann cells cultured from *LPA1*-null mice exhibit greatly diminished morphological responses to LPA (Weiner et al., 2001). Intrathecal injection of LPA induced morphological, biochemical and behavioral changes similar to those observed after nerve injury. However, mice lacking the LPA1 receptor do not develop signs of neuropathic pain after peripheral nerve injury, suggesting that receptor-mediated LPA signaling is crucial in the initiation of neuropathic pain (Inoue et al., 2004). Although there is no direct, accurate evidence characterizing the role of Schwann cell LPA1 receptors in neuropathic pain, the LPA1 receptor is a critical factor in the mediation of neuropathic pain by Schwann cells.

Hydroxyl carboxylic acid receptor type 2 (HCAR2), another class of GPCR is primarily expressed on adipocytes, peripheral immune cells and brain microglia, and involved in lipogenesis, inflammatory processes (Offermanns, 2017). Recently, the HCAR2 expression has been highlighted in the sciatic nerve, primarily in cells positive for S100 specifically marked by Schwann cells. An up-regulation of HCAR2 in the sciatic nerve and the DRG is present in neuropathic mice. Moreover, the HCAR2 endogenous ligand β-hydroxybutyrate can reduce the tactile allodynia in neuropathic pain models in female but not male mice. But the effect only occurs in wild-type mice but not in the HCAR2-null mice (Boccella et al., 2019). Although HCAR2 is not specifically expressed in Schwann cells, and do not have the evidence to investigate its role in conditional knockout mouse models, HCAR2 may be a new receptor for the regulation of neuropathic pain.

γ-aminobutyric acid type B (GABA-B) receptor, is one of the native targets of GABA, and mediates the inhibitory transmission in the CNS. Emerging evidence of GABA-B receptor functions in the PNS suggest its contribution in regulating maturation and plasticity of Schwann cells (Magnaghi et al., 2006). Both GABA-B1 and GABA-B2 receptors are present in neurons, and also in Schwann cells (Magnaghi et al., 2004). The conditional mice with specific deletion of GABA-B receptors in Schwann cells are hyperalgesic and allodynia, which associated with a morphological phenotype characterized by a peculiar increase in the number of small unmyelinated fibers and Remak bundles, including nociceptive C-fibers (Procacci et al., 2012; Faroni et al., 2014a). Meanwhile, GABA-B receptor activation following GABA-B ligands treatments with baclofen and CGP56433, promotes nerve regeneration and ameliorates neuropathic pain (Magnaghi et al., 2014). These findings support the importance of GABA-B receptors in the peripheral myelination process, and in modulating the nociceptive fiber activity.

The class II major histocompatibility complex (MHC-II) is presented on the surfaces of antigen presenting cells (APCs) for recognition by T cells. The main professional APCs are dendritic cells, macrophages and B cells (Neefjes et al., 2011). However, under traumatic and inflammatory conditions, Schwann cells have the potential to express MHC-II and present antigens (Meyer zu Hörste et al., 2010a,b). Notably, Schwann cells upregulate MHC-II under traumatic conditions in female but not male rats (Liu et al., 2012). MHC-II on Schwann cells activates T helper cells and promotes posttraumatic axon loss and subsequent neuropathic pain. Meanwhile, deletion of MHC-II in myelinating Schwann cells can diminish mechanical allodynia and thermal hyperalgesia in a chronic constriction injury (CCI) model in female mice (Hartlehnert et al., 2017).

Other interesting examples suggest that Schwann cells directly control the development of neuropathic pain. Deficiency of the Neuregulin/ErbB cell signaling pathway induces an enhanced response to mechanical allodynia but no changes in thermal hyperalgesia (Chen et al., 2006). In addition, mice expressing the HIV-1 envelope glycoprotein gp120 in nonmyelinating Schwann cells and treated with didanosine, an antiretroviral drug, had changes in sensory function (thermal allodynia; Keswani et al., 2006). Another candidate target is the acetylcholine system. *Botulinum* neurotoxins, an inhibitor of acetylcholine neurotransmitter release, exerting their action by cleaving soluble NSF attachment proteins, SNARE proteins, is well established in the treatment of neuropathic pain (Oh and Chung, 2015). *In vitro* experiments showed that *Botulinum* neurotoxins was able to interact with the proliferative state of Schwann cells, and facilitate Schwann cell proliferation (Marinelli et al., 2012). Moreover, acetylcholine receptors are present on Schwann cell membrane, and the activation of acetylcholine receptors can induce myelin structure reorganization (Verdiyan et al., 2016). Although there is no direct evidence to strongly characterize the role of Schwann cell acetylcholine system in neuropathic pain, these studies indicate that *Botulinum* neurotoxins may regulate the function of Schwann cells by the acetylcholine system to reduce neuropathic pain.

## Regulation of Growth Factors, Cytokines, and Chemokines in Schwann Cells in Neuropathic Pain

In response to nerve injury, glial mediators produced or released by activated Schwann cells or recruited immune cells are also a key issue in the control of neuropathic pain by Schwann cells. As shown in Table 2, Schwann cells produce both large molecules, such as growth factors, cytokines and chemokines, and small molecules, including ATP. These mediators from Schwann cells play a critical role in neuronal and synaptic activity as well as pain.

Proinflammatory cytokines such as TNF-α, IL-1, and IL-6 are well-studied glial mediators. The expression levels of these molecules are upregulated in the sciatic nerve, spinal cord and DRG of animals after CCI (Martucci et al., 2008). Activated Schwann cells and infiltrating macrophages release these mediators, which contribute to axonal damage and enhance nociceptor activity (Campana, 2007; Sacerdote et al., 2008). TNF, the most prominent proinflammatory cytokine, is detectable as early as 6 h after sciatic nerve injury but has no significant difference from the control group within 7 days after nerve injury (Scholz and Woolf, 2007). Activated resident Schwann cells and macrophages are thought to be the sources for TNF production in the early stage of sciatic nerve injury (Campana, 2007; Sacerdote et al., 2008). IL-1 and IL-6 are also produced at the onset of injury but have a sustained effect on neuropathic pain. In CCI models, the mRNA levels of these cytokines increase at 1 day after injury, and the levels remain high for up to 21 days after surgery. In addition to the proinflammatory cytokines, anti-inflammatory mediators are also critical to the regulation of neuropathic pain (Martucci et al., 2008). The accumulating reports show that IL-10, the predominant anti-inflammatory mediator, is detected in the sciatic nerve after a lesion develops (Austin and Moalem-Taylor, 2010; Franchi et al., 2012). Meanwhile, several strategies aimed at enhancing the level of endogenous IL-10 have succeeded in preventing and relieving pain hypersensitivity in several neuropathic pain models (Franchi et al., 2012; Wang et al., 2012).

Erythropoietin (Epo), another anti-inflammatory cytokine, is upregulated along with its receptor EpoR after nerve injury, and the primary cellular source of EPO is activated Schwann cells (Li et al., 2005). In the early stage of nerve injury, Epo is able to reduce the production of TNF-α and facilitate recovery from chronic pain states (Keswani et al., 2004; Campana et al., 2006b). Therefore, the balance of inflammatory and anti-inflammatory mediators may be a future target for therapeutic intervention.

Chemokines, as another pain modulator, are expressed in neurons as well as in glial cells (Ji et al., 2013, 2016). In Schwann cells, cyclooxygenase-2 (COX-2) and monocyte chemoattractant protein-1 (MCP-1) are expressed after nerve injury (Toews et al., 1998; Takahashi et al., 2004). However, the cellular sources of these molecules differ between the early and late stages of nerve injury. The first increase in the number of positive cells occurred approximately 1 day after nerve injury in Schwann cells coexpressing S-100. The second increase was noted after 7–14 days and these cells were macrophages coexpressing ED-1 (Toews et al., 1998; Takahashi et al., 2004). Notably, a distinct CCL2 and TRPA1/oxidative stress pathway induced macrophage accumulation in Schwann cells (De Logu et al., 2017). Oxidative stress has been thought to exert a chemoattractant effect by macrophage infiltration. NADPH oxidase-1 (NOX1) is noticeably expressed in Schwann cells. TRPA1 in Schwann cells activates a NOX1-mediated intracellular signaling pathway to produce sustained oxidative stress, and it also maintains macrophage infiltration into lesion sites, which induces allodynia by activating TRPA1 on the nociceptors (De Logu et al., 2017).

Growth factors are known to play important roles in neuronal survival, myelination and synaptic plasticity (Park and Poo, 2013). In response to sciatic nerve injury, Schwann cells secrete multiple growth factors, such as NGF, BDNF, and neurotrophin-3 and 4 (Scheib and Höke, 2013). Among these factors, we found that TNF-α-induced BDNF release beyond the basal level in Schwann cells depends on P2X4R (Su et al., 2019). However, P2X4R-knockout mice do not show pain hypersensitivity after nerve injury, which has an ill effect on the release of intracellular signaling factors, including BDNF (Tsuda et al., 2003). On the other hand, as BDNF is a key mediator of neuropathic pain, BDNF-knockout mice display reduced pain hypersensitivity after nerve lesion compared with wild-type mice (Yajima et al., 2005). However, overexpression of P2X4R in Schwann cells did not result in increased pain hypersensitivity. In addition, intraplantar injection of BDNF induced mechanical allodynia only in naïve mice and not in mice with crush injuries of the sciatic nerve (Su et al., 2019).

ATP is an important intracellular messenger molecule that interacts with purinoceptors, playing a crucial role in the formation and regulation of neuropathic pain (Burnstock, 2006). Several subtypes of P2X receptors are highly expressed in Schwann cells (Su et al., 2019). ATP is released from Schwann cells under physiological conditions, and after nerve injury, increasing levels of ATP play an important role in both peripheral and central sensitization (Martucci et al., 2008; Tsuda et al., 2010). Released ATP activates neurons and Schwann cells to release certain mediators, including proinflammatory cytokines and chemokines (Inoue, 2006). Notably, when the broad-spectrum P2 receptor antagonist pyridoxal phosphate-6-azophenyl-2′,4′-disulfonic acid (PPADS) was administered after nerve injury, it significantly reduced the levels of IL-1 and IL-6, decreased tactile allodynia and thermal hyperalgesia, and continued to exert an effect for 2–3 weeks after administration (Martucci et al., 2008).

## Relationship of Demyelination and Remyelination With Neuropathic Pain

It is well established that demyelination contributes to the development of neuropathic pain by disrupting the precise molecular and structural features of nerve fibers. For example, in females specifically, algesic MBP fragments released from the intact myelin sheath after nerve injury *via* MT1-MMP proteolysis control mechanical allodynia (Hong et al., 2017). The alteration of ErbB signaling in myelination Schwann cells leads to demyelination and induces mechanical hypersensitivity (Tao et al., 2013).

However, a direct relationship between remyelination and pain relief has not been investigated. Several studies have shown an indirect association between them. For example, Gabapentin alleviates mechanical and thermal allodynia and improves nerve remyelination after chronic constriction of the sciatic nerve (Camara et al., 2015). Focal lysolecithin-induced demyelination of peripheral afferents results in neuropathic pain behaviors, which are reversible by cannabinoids after nerve remyelination nearly 3 weeks after treatment (Wallace et al., 2003). Our work reveals that the P2X4-LV group is related to improve remyelination and has the potential to reduce the mechanical allodynia induced by sciatic nerve injury (Su et al., 2019). Thus, the accurate relationship of remyelination with neuropathic pain will require further study.

## Conclusions

Peripheral neuropathic pain is a disorder caused by nerve trauma or disease. In this review article, we mainly discuss the role of Schwann cells in the development and relief of neuropathic pain directly induced by nerve injury. In fact, diabetes may induce several types of neuropathies, resulting in spontaneous pain and eventual loss of pain sensation (Mizisin, 2014; Gonçalves et al., 2017). The state of Schwann cells and their communication with axons might be disturbed, ultimately leading to fiber loss and pain. However, the mechanistic understanding of the Schwann cell response to diabetes is unclear. In addition to the importance of glial receptors and mediators, Schwann cell autophagy represents a powerful approach to prevent the onset and chronification of neuropathic pain (Marinelli et al., 2014). Notably, caloric restriction promotes Schwann cell autophagy *via* AMP-activated protein kinase and facilitates remyelination in nerve injury, which provides new evidence for Schwann cell autophagy as a therapeutic approach against neuropathic pain (Coccurello et al., 2018). Overall, accumulating evidence has revealed the key role of Schwann cells in the regulation of neuropathic pain. An improved and extended comprehension of the underlying neurobiological mechanisms of neuropathic pain would allow the development of successful targeted pain therapy.

## Future Prospects

Increased mechanistic study of the response of Schwann cells to nerve injury could reveal the underlying molecular mechanisms specific to neuropathic pain. The first pertinent approach would be to expand the recent development of conditional knockin/knockout mouse models to study the specific roles of target genes expressed in Schwann cells following nerve injury. Second, nonmyelinating Remak Schwann cells are characterized by a lack of myelin, but they also characteristically express cell surface receptors and cell adhesion molecules that are downregulated on myelinating Schwann cells. The difference between the two types of Schwann cells is a factor that merits additional study in the future. Third, neuropathic pain can result from different types of nerve injury or disease but has a higher prevalence in females than in males. How sex differences influence neuropathic pain must also be elucidated in future studies. Finally, drug targeting or transplantation of Schwann cells will be of great interest in the search for therapeutic strategies against neuropathic pain.

## Author Contributions

GC and ZW contributed to the conception and design of the review and wrote the first draft of the manuscript. ZW, GC, WS and YF wrote sections of the manuscript. All authors contributed to manuscript revision, read and approved the submitted version.

## Conflict of Interest Statement

The authors declare that the research was conducted in the absence of any commercial or financial relationships that could be construed as a potential conflict of interest.
